# Supplementary material for: Splice-Junction-Based Mapping of Alternative Isoforms in the Human Proteome
Source: Cell Rep. Author manuscript; Available in PMC 2020 Jan 15. (PMC6961840; doi:10.1016/j.celrep.2019.11.026)
Supplement: 2 [file NIHMS1546469-supplement-2.zip › DF1/PXD006675/LeftAtrium/LeftAtrium_5_CLTCL1_LQEHFQLQNLGINPANIGFSTLTMESDK.pdf]

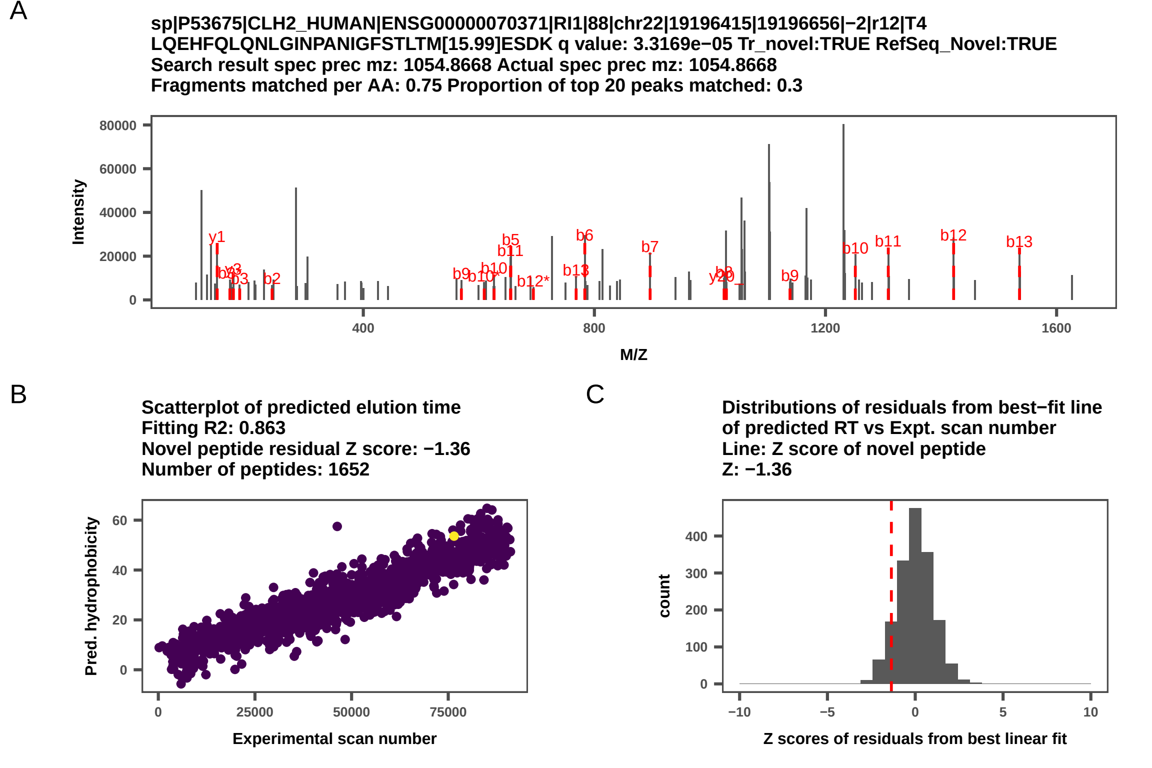
A
sp|P53675|CLH2_HUMAN|ENSG00000070371|RI1|88|chr22|19196415|19196656|−2|r12|T4
LQEHFQLQNLGINPANIGFSTLTM[15.99]ESDK q value: 3.3169e−05 Tr_novel:TRUE RefSeq_Novel:TRUE
Search result spec prec mz: 1054.8668 Actual spec prec mz: 1054.8668
Fragments matched per AA: 0.75 Proportion of top 20 peaks matched: 0.3
Intensity
80000
60000
40000
20000
0
y1
b2
b5
b11
b6
b7
b9
b10
b11
b12
b13
b12*
M/Z
400
800
1200
1600
B
Scatterplot of predicted elution time
Fitting R2: 0.863
Novel peptide residual Z score: −1.36
Number of peptides: 1652
Pred. hydrophobicity
60
40
20
0
25000
50000
75000
Experimental scan number
C
Distributions of residuals from best−fit line
of predicted RT vs Expt. scan number
Line: Z score of novel peptide
Z: −1.36
count
400
300
200
100
0
−10
−5
5
10
Z scores of residuals from best linear fit
